# Detecting Genetic Mobility Using a Transposon-Based Marker System in Gamma-Ray Irradiated Soybean Mutants

**DOI:** 10.3390/plants10020373

**Published:** 2021-02-15

**Authors:** Nguyen Ngoc Hung, Dong-Gun Kim, Jae Il Lyu, Kyong-Cheul Park, Jung Min Kim, Jin-Baek Kim, Bo-Keun Ha, Soon-Jae Kwon

**Affiliations:** 1 Advanced Radiation Technology Institute, Korea Atomic Energy Research Institute, Jeongeup 56212, Korea; nguyenhung@kaeri.re.kr (N.N.H.); dgkim@kaeri.re.kr (D.-G.K.); jaeil@kaeri.re.kr (J.I.L.); jmkim0803@kaeri.re.kr (J.M.K.); jbkim74@kaeri.re.kr (J.-B.K.); 2 Department of Applied Plant Science, College of Agriculture and Life Science, Chonnam National University, Gwangju 61186, Korea; 3 Department of Life-Resources, Graduate School, Sunchon National University, Sunchon 57922, Korea; 4 Department of Agriculture of Life Industry, Kangwon National University, Chuncheon 24341, Korea; kyongcheul.park@kangwon.ac.kr

**Keywords:** soybean, transposable element, mutation breeding, gamma ray, TE-TRAP

## Abstract

Transposable elements (TEs)—major components of eukaryotic genomes—have the ability to change location within a genome. Because of their mobility, TEs are important for genome diversification and evolution. Here, a simple rapid method, using the consensus terminal inverted repeat sequences of PONG, miniature inverted-repeat transposable element (MITE)-Tourist (M-t) and MITE-Stowaway (M-s) as target region amplification polymorphism (TE-TRAP) markers, was employed to investigate the mobility of TEs in a gamma-irradiated soybean mutant pool. Among the different TE-TRAP primer combinations, the average polymorphism level and polymorphism information content value were 57.98% and 0.14, respectively. Only the PONG sequence separated the mutant population into three major groups. The inter-mutant population variance, determined using the PONG marker (3.151 and 29%) was greater than that of the M-t (2.209 and 20%) and M-s (2.766 and 18%) markers, whereas the reverse was true for the intra-mutant population variations, with M-t and M-s values, being 15.151 (82%) and 8.895 (80%), respectively, compared with the PONG marker (7.646 and 71%). Thus, the MITE markers revealed more dynamic and active mobility levels than the PONG marker in gamma-ray irradiated soybean mutant lines. The TE-TRAP technique associated with sensitive MITEs is useful for investigating genetic diversity and TE mobilization, providing tools for mutant selection in soybean mutation breeding.

## 1. Introduction

Soybean (*Glycine max* L.) is an agriculturally important leguminous crop worldwide.

Soybean seeds are rich in seed protein (average 40%) and oil (average 20%). This composition is valuable for a variety of human and animal consumption applications, such as feed, biodiesel, edible oils and other food products [1]. Soybean is also frequently cultivated and consumed directly by humans, having been part of the Asian diet for several centuries due to its nutritional and health benefits [2,3]. Furthermore, soybean is used for important industrial purposes, including biofuel, hygienics and cosmetics. Approximately 98% of soybean meal is used as livestock and aquaculture feed because of its composition, including high quality protein, a low saturated fat level and no cholesterol [4]. Soybean also supplies a remarkable level of additional nitrogen to the soil, allowing for diversified crop rotations and promoting the production of other crops [5].

Mutation breeding in crop plants has been effective in improving important agronomic traits. However, the practical use of varieties resulting from spontaneous mutations is still arduous because of the extensive selection progress and low mutation rates of only 10^−5^–10^−8^ per generation [6]. Gamma-ray radiation breeding has become a very effective method of inducing mutations in seeds and other planting materials, such as cuttings, pollen or tissue-cultured calli [7,8], and directly produces mutant varieties [9]. Through the application of high-throughput genomic sequencing, gamma-ray radiation has been shown to induce DNA damage, including deletions, duplications, inversions and translocations of all sizes [10]. Soybean mutants resulting from gamma-ray radiation have been shown to have enhanced agronomic traits (yield and flowering time) [11], nutritional qualities (phytate levels and lipoxygenase-free seeds) [12,13] and increased abiotic stress tolerance (germination in dry or wet climates and flood tolerance) [14,15].

Transposons, or transposable elements (TEs), which are mobile genetic elements, constitute a large fraction of eukaryote genomes and are able to relocate within a genome [16]. TEs play important roles in genome evolution and diversification by regulating the expression of adjacent genes [17] and rearranging plant genomes and epigenomes [18,19]. The transposition of these elements contributes to genomic plasticity by enhancing various chromosomal mutations and increasing allelic diversity [20,21]. On the basis of transposition mechanisms, TEs are conventionally categorized into two classes: Class I (RNA) elements, or retrotransposons that transpose via RNA intermediates, and Class II (DNA) elements, that transpose via DNA intermediates [22]. Class II TEs are categorized into several subclasses based on similarities in terminal inverted repeat sequences (TIRs) and target site duplications (TSDs). Miniature inverted-repeat TEs (MITEs) and PONG are the two most abundant families of the Class II DNA transposons in the soybean genome [23]. Unlike other Class II TEs, MITEs are small TEs (usually <500 bp) but are present in very high copy numbers in the genome, contain short TIR sequences and lack coding-gene capacity. Most of the 10,000 plant MITEs have been further divided into two major groups—Tourist-like (M-t), with TAA as the TSD, and Stowaway-like (M-s), with TA as the TSD—and several other minor groups. The soybean genome has undergone multiple whole-genome level duplications [24], making it one of the most complex plant genomes investigated to date [25]. This complexity, in addition to the dynamic activities (inactivation and reactivation) of TEs in the genome, has resulted in limited research being conducted on soybean genomics [25].

The genome-wide distributions of TEs allows them to be used as molecular markers for the estimation of genetic diversity and structural variations in various plant genomes, in conjunction with molecular techniques for TE detection, such as amplification fragment length polymorphisms (AFLP) in rice [26,27], transposon display (TD) in rice [28], maize [27,29], Arabidopsis and Brassica [30], sequence characterized amplified region (SCAR) in maize [29] and TE-based target region amplification polymorphisms (TE-TRAP) in sorghum [31]. The TE-TRAP marker system is a modification of TRAP that is a relatively new, simple and powerful method for dissecting genetic variation [32]. The conventional TRAP marker system has been applied successfully in the dissection of genetic variation in many crops, and it has recently been used for detecting DNA mutations [33]. In this study, the TRAP system was modified to develop a TE-TRAP marker system using MITE and PONG TE subfamily sequence information. The TIR sequences of representatives of the Class II transposon family have been used to develop fixed primers associated with arbitrary primers that targeted intron or exon regions with AT- or GC-rich cores to amplify fragments. Kikuchi et al. [34] suggested that miniature Ping, which belongs to the MITE family, is activated under stress conditions, such as physical mutagenesis with gamma rays. Here, we used the newly described TE-TRAP marker technique with MITE and PONG sequence information. The objective of our study was to investigate genetic diversity and transposon mobility, as assessed by polymorphisms generated by MITE and PONG, among irradiated soybean mutant lines.

## 2. Results

### 2.1. Numbers of Amplicons and Polymorphisms among the TE-TRAP Markers

In the previous study by Kim et al. 2020, seeds of eight soybean cultivars (as mentioned in the material and methods section) were irradiated and mutant populations were constructed through 12 generations to reach 208 genetically fixed mutant lines (201 mutant associated with their original cultivars). Their usefulness for mutation breeding was determined by the TRAP marker. In this study, to characterize the interactions of transposon mobility in gamma ray mutant populations, the genetic diversity of 208 mutant diversity pool (MDP) lines were compared using the TE-TRAP marker. TE-TRAP was performed with 12 primer combinations, including three fixed forward primers (M-t, M-s and Pong) designed based on the TIR sequences of TEs, in combination with arbitrary reserve primers Sa4, Sa12, Ga3 and Ga5, which were provided by a previous study from G.Li and C.Quiros et al. 2001 [35] and Hu et al. 2005 [36]. The TE-TRAP produced high amplification profiles. The numbers of amplicons, percentages of polymorphisms and polymorphic information content (PIC) values among mutant diversity pool (MDP) lines are shown in Table 1 and Table 2. In total, the 12 primer combinations amplified 407 fragments, ranging from 22 (PONG + Ga5) to 47 (M-t + Sa4) amplicons per primer combination, at sizes of 100–1000 bp. Of the 407 amplicons scored, 170 (37.9%) were monomorphic and 237 (58.00%) were polymorphic. An average of 33.92 amplicons with 19.75 polymorphic fragments was scored per primer combination. The highest level of polymorphism (77.42%) was obtained from the primer combination PONG + Sa4, whereas the lowest level of polymorphism was obtained from the primer combination M-t + Sa4 (38.46%) (Table 1). The PIC values of M-s, M-t and PONG had averages of 0.12, 0.14 and 0.15, respectively, ranging from 0.09 (M-t + Sa4 and M-s + Ga5) to 0.20 (PONG + Sa4) (Table 1). The number of fragments consistently revealed that MITE markers, including M-t and M-s, generated more fragments than the PONG marker; however, the polymorphism rate (%) and PIC values determined using the PONG marker were greater than those determined using the M-t and M-s markers (Table 2).

### 2.2. Genetic Differentiation

**Figure 1:**
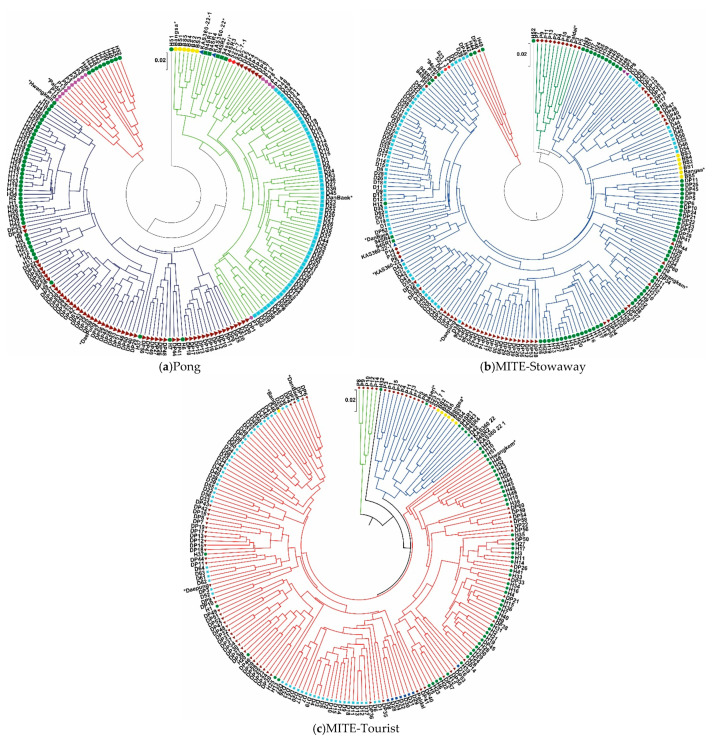
Dendrograms revealed by unweighted pair group method with arithmetic mean cluster analyses and the population structure of soybean MDP lines based on target region amplification polymorphism (TE-TRAP) markers (**a**) PONG; (**b**) miniature inverted-repeat transposable element (MITE)-Stowaway, and (**c**) MITE-Tourist. * Indicates original cultivars. Mutant line abbreviations are based on the names of the original cultivars. HK and BS populations are indicated with green and yellow circles, respectively, 94Seori and DB populations are indicated with green and blue squares, respectively, KAS360 and DP populations are indicated with blue and brown triangles, respectively, and P and 527 populations are indicated with pink and red quadrangles, respectively.

**Figure 2:**
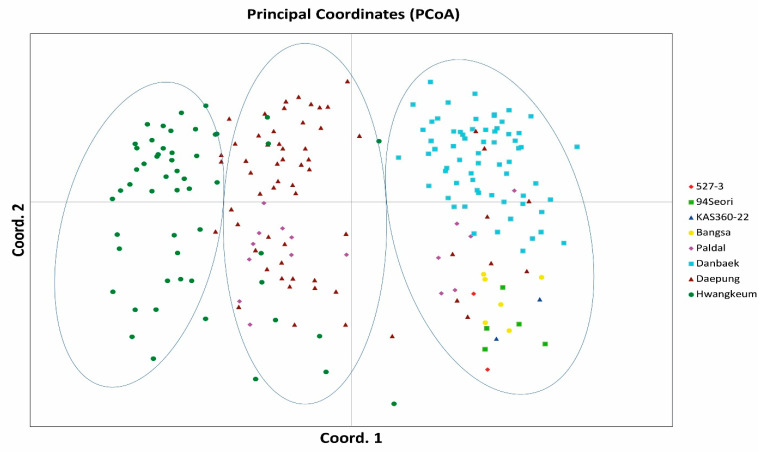
Two-dimensional principal component analysis ordination of MDP mutant lines based on Pong TE-TRAP marker diversity.

On the basis of genetic distances, dendrograms were constructed to identify the genetic relationships among the 208 MDP lines (Figure 1a–c). For all three TE-TRAP markers, M-t, M-s and PONG, the seven wild-type cultivars and their mutants could be divided into three major groups. In the cluster pattern based on the PONG marker, the first group comprised Bangsa (BS) and BS mutants, 94Seori and 94Seori mutants, KAS360-22 and its mutants, Danbaek (DB) and DB mutants, and several mutants from both Daepung (DP) and Paldal (P). The second group contained DP and DP mutants, and Hwangkeum (HK) and HK mutants. The third group contained P and P mutants, and nine lines originating from HK. In the cluster pattern based on M-s, the first group comprised P and P mutants. The second group contained HK and HK mutants, DP and DP mutants, BS and BS mutants, KAS360-22 and its mutants, and 523-7 and its mutants. The third group contained two DP mutants, three HK mutants and one DB mutant. In the cluster pattern based on M-t, the first group contained P and five P mutants, and the second group contained 94Seori and 94Seori mutants, BS and five BS mutants, and both KAS360-22 and 523-7 and their mutants. The third group contained HK and HK mutants, DB and DB mutants, and DP and DP mutants. In the cluster analysis, the M-t and M-s clustering patterns did not clearly partition the DB, DP and HK groups, in which a few mutants did not correspond precisely to the wild-type cultivars. Principal coordinate analyses (PCoAs) were performed to provide further insights into the genetic differences among soybean MDP lines. The PCoA results corresponded well with the cluster patterns produced by Pong (Figure 2) and M-t and T-s (Figure S1). In the PCoA plot for PONG, three distinct groups were separated. The mutants and their original cultivars were distributed closely and, except a few individuals, were separated from the clusters. In contrast, when compared with the characteristic high consensus of the PONG-based dendrogram, discrepancies when forming subgroups within the major groups from M-t and M-s were noted. Unstable groupings were seen in the PCoA plots produced by M-t and M-s. KAS-360, BS and their mutants were clearly separated, but the other three cultivars (HK, DP and DB) and their mutants clustered together in several subgroups.

### 2.3. Analysis of Molecular Variance (AMOVA)

An AMOVA based of the TE-TRAP marker system using permutational testing procedures was performed to determine and separate the total molecular variance into among-population variance (variance caused by different mutant populations) or within-population variance (variance caused by differences between wild-type and mutants) and to support the results of the dendrogram and PCoA of each marker. The AMOVA showed estimated among-population variances of 3.151 (29%), 2.209 (20%) and 2.766 (18%) using PONG, M-s and M-t, respectively, whereas the estimated within-population variances were 7.646 (71%), 8.957 (80%) and 12.385 (82%) using PONG, M-s and M-t, respectively (Table 3). Thus, the majority of variance was derived from within populations for PONG, M-t and M-s. The results of the AMOVA of MDP soybean lines and the TE-TRAP marker system showed that the estimated among-population variation value of PONG, at 3.15 (29%), was greater than those of M-s, at 2.209 (20%), and M-t, at 2.766 (18%). In contrast, the estimated within-population variation value using the PONG marker, at 7.646 (71%), was less than those of M-s, at 8.957 (80%), and M-t, at 12.315 (82%). Thus, a greater percentage of variation within groups might be observed in the M-t and M-s data than in the PONG data.

## 3. Discussion

Transposable elements account for large portions of the genomes of major crops, including 40% of *Oryza sativa* [37], >80% of *Zea mays* [29] and 50% of soybean [24]. Class II TEs have been successfully adopted in soybean genetic investigations in recent decades [38]. However, our understanding of the active mechanisms and mobility of TEs in plants is still largely lacking. Using tagged DNA TEs as molecular markers to develop a TE-based marker system will allow a multitude of mutations to be detected.

To dissect the genetic relationships among 208 MDP mutant soybean lines, we evaluated DNA polymorphisms and genetic differentiation using a TE-TRAP marker system. In this study, the TE-TRAP markers, with 12 primer combinations, generated 407 fragments from 208 MDP soybean mutant lines, and there was considerable variation in the percentages of polymorphic fragments amplified by primer combinations, ranging from 56 (PONG + Sa12) to 77.42% (PONG + Sa4), with an average of 58%. The PIC values of the TE-TRAP markers ranged from 0.09 to 0.20, with an average of 0.14. The MITEs are Class II TEs that have very high copy numbers in the soybean genome, with an estimated 1575 and 1758 copies of M-t and M-s, respectively [23]. In the TE-TRAP marker system, M-t and M-s produced large numbers of amplified bands and high levels of polymorphism (Table 1 and Table 2). PONG has only 102 copies in the soybean genome [23]. Thus, the PONG primers did not produce as many resolvable bands as the MITE markers, but the PONG marker generated a large number of polymorphic fragments and a greater level of polymorphism (61.46%). In a previous study of sorghum, Im et al. [31] obtained 1133 fragments using 31 primer combinations, with a mean value of 36.5 per primer combination. The PIC value of the TE-TRAP in sorghum is 0.172. When comparing the results of the TE-TRAP with the conventional TRAP marker system, using MDP mutant lines, which were constructed by Kim et al. [39], the latter obtained an average 59% polymorphism level and an average of 34.44 fragments. In the present study, we observed a 58% polymorphism level and an average of 33.92 fragments. In another study using the TRAP marker system with a gamma-irradiated faba bean mutant population, Lee et al. [33] obtained an average 66.7% polymorphism level and an average of 20.1 fragments. The overall amplification profile of the TE-TRAP was quite similar to that of the conventional TRAP technique. These results confirm that the TE-TRAP marker system is able to detect many functional loci in few reactions and is highly efficient in identifying the diversity level in radiation-based breeding.

The constructed dendrograms, based on the unweight pair group method with arithmetic mean analysis using TE-TRAP marker data, revealed the relationships among the mutant lines. Overall, only the clustering pattern of the PONG marker separated the lines into three major groups, whereas those of the M-t and M-s markers did not show clear distinctions between mutants and original cultivars. In a previous genetic diversity analysis of an MDP mutant population using the TRAP marker system, MDP lines clustered into four major groups that largely corresponded to their wild-type cultivars and pedigree data [39]. These different patterns revealed by TRAP and TE-TRAP, markers indicate that the TE-TRAP technique using TIR consensus sequences of M-t and M-s could detect a great variability in the irradiated soybean mutant population. Inconsistencies have also been observed in other TE-based marker systems, such as MITE–AFLP [40,41] and CACTA-TD [42], as well as from physical mutagenesis, such as x-, ion- and gamma-ray irradiation [43,44]. The TE activities induced by some molecular techniques in a genome might not completely cover the diversity existing at the DNA level, mainly owing to the TE-mediated intraspecific violation of genetic collinearity and gene structural variations existing in plant species, such as in maize [45,46,47]. Furthermore, owing to complex duplicated genome structures (polyploidy), TE activity and MITE characteristics (approximately 500 bp in length), next-generation sequencing, with its short reading length capacity (200–250 bp in length), cannot efficiently produce a near-accurate genome sequence or transposition mechanisms in soybean mutant populations [24,25]. Thus, molecular markers based on TEs may be more efficient in capturing molecular genetic diversity owing to transposition events and in creating allelic diversity through insertional polymorphisms in TEs [48]. Additionally, some polymorphisms and genetic diversity might result from TE mobilization, and MITE-based markers may produce allelic diversity at several loci through insertional polymorphisms after exposure to a physical mutagen, such as gamma-ray irradiation.

The AMOVA revealed that the major proportion of the genetic diversity was from within-population variation using the three different markers (Table 3). This indicates that molecular variation induced by TEs in irradiated mutant populations is attributed to differences within individual populations and corresponds with results of the AMOVA analysis using the TRAP marker system on MDP lines [39]. The percentage of estimated among-population variation as assessed by the PONG marker (29%) was greater than those assessed by M-t (20%) and M-s (18%), indicating that genetic diversity was induced in the loci amplified by the PONG primers. However, the estimated within-population variation, as determined by M-s (80%) and M-t (82%), was greater than that determined by PONG (71%), indicating that random variation occurred more often in individual lines of each mutant sub-population (between wild-type and mutants). This tendency was similar to that observed in the clustering pattern and the PCoA results of M-t and M-s (Figure 1 and Figure S1). In the same irradiated MDP mutant population, the general clustering patterns from TRAP [39] and from TE-TRAP, using consensus TIR sequences of PONG, were similar, with the exception of pedigree lineages. However, TE-TRAP using consensus TIR sequences of M-t and M-s showed greater discrepancies in the placement of a few mutant lines that did not group with the wild-type. Thus, MITEs might be dynamic and their sensitivity affected in the gamma-ray irradiated mutant population. The present results also suggest that, compared with PONG, M-t and M-s markers revealed more mobile TEs in the genome, and therefore, are potential markers for investigating molecular characteristics in mutant populations. This hypothesis is similar to those of several studies investigating heterosis of inbred lines using MITE insertional polymorphism marker systems in outbreeding programs, such as DcMaster transposon display in carrot [49], MITE marker amplification in snapdragon [50] and MITE-AFLP markers in maize [42]. TE markers are derived from low-copy or protein-coding regions [37,51]. MITE-transposon display produces high allelic variation in segregating populations of rice and maize [52]. As seen in Kikuchi et al. [34], rice MITEs are effectively mobilized under stress conditions, including tissue culturing or in response to gamma-ray irradiation, suggesting that such transposition events account, in part, for the high mutation frequency of rice [34]. Thus, specific MITE-based markers might provide new insights into studies of genetic diversity and help breeders select for better mutant lines of important crop species, including irradiated mutant soybean populations.

## 4. Materials and Methods

### 4.1. Plant Materials and Genomic DNA Extraction

In total, 208 gamma-ray irradiated soybean mutant lines formed the MDP. The MDP was constructed using the 12th generation of seven irradiated soybean cultivars, the soybean landrace KAS360-22 and six Korean soybean cultivars—94Seori, BangSa (BS), PalDal (P), DanBaek (DB), DaePung (DP) and HWangKeum (HK)—obtained in a previous experiment by Kim et al. [39]. The genomic DNA was extracted using a DNeasy 96 Plant Kit (Qiagen, Leipzig, Germany). The extracted DNA was quantified using a Nano Drop Spectrophotometer (Thermo Fisher Scientific, Walthan, MA, USA) and then, the concentrations were adjusted to 10 ng/µL.

### 4.2. TE-TRAP Analysis

For the fixed primer design, the TEs sequence analysis and the progress of primer design is described as flowchart in Figure S2. The representative soybean MITE sequences: Tourist, Stowaway and PIF/Pong were identified in whole soybean genome sequences from the SoybaseTEdb (http://soybase.org/Soytedb accessed on 21 February 2021). MITE groups with more than one hundred members were retrieved against BLAST program supporting in the SoyTEdb. Through this procedure, nucleotide sequences of specific TIR regions of each MITEs were discovered and aligned using a multiple sequence alignment program (MAFFT) [51]. The degenerate primers were designed to match 19–20 bp sequences corresponding to the 20 bp TIRs of the M-s, M-t and PONG elements, from the conserved region covering all nucleotide sequences of each soybean MITEs. The fixed primers were designed manually against selected TIRs of the TE sequences using the web-based PCR primer-design software Primer3 (http://www-genome.wi.mit.edu/cgi-bin/primer/primer3.cgi accessed on 21 February 2021).

Three fixed primers and four arbitrary primers were used to generate TE-TRAP markers (Table 4). TE-TRAP amplifications with 12 primer combinations were carried out on all the DNA samples in accordance with the protocol of Hu et al. [35] with slight modifications. Briefly, reactions were performed in 20 µL volumes containing 2 µL genomic DNA (10 ng/µL), 1 µL fixed primer, 1 µL of each arbitrary primer (10 pmol/µL), 0.4 µL of dNTPs (10 mM), 2.0 µL 10× PCR buffer and 0.3 µL Phoenix Taq DNA polymerase (5 U/µL; cat no. Phoenix2013). The PCR amplification was performed by initially denaturing template DNA at 94 °C for 2 min, followed by five cycles of 94 °C for 45 s, 35 °C for 45 s and 72 °C for 60 s, then 35 cycles of 94 °C for 45 s, 53 °C for 45 s and 72 °C for 60 s, and a final extension at 72 °C for 7 min to terminate the reaction. The amplified products were analyzed separately using a fragment analyzer automated capillary electrophoresis instrument (FA; Advanced Analytical Technologies, Ankeny, IA, USA), and the collected images were scored manually.

### 4.3. Data Analyses

Each amplified fragment was scored with binary data (1 or 0 for presence or absence, respectively). Using a 0.1-matrix, we calculated the gene diversity, mono and polymorphic percentages, PIC value and genetic distance using the genetic analysis package Power Marker [52]. A dendrogram was constructed using the unweight pair group method with arithmetic mean algorithm based on the Nei’s distance method [53] in Power Marker 3.2.5 associated with MEGA X. To estimate the genetic relationships among the 208 MDP soybean lines, we performed a PCoA using GenALEx v6.502. We then conducted an AMOVA and calculated genetic distances to support the genetic diversity information. An AMOVA of 999 permutations was completed to assess inter- and intra-population variance (wild-type and mutants) using GenALEx v6.502 [54].

## 5. Conclusions

In this study, the overall amplification of MITEs was significant enough to confirm their practical utility as molecular markers for investigating mutant populations, after having induced random variation, such as that resulting from physical mutagenesis (X-ray or gamma-ray). We also demonstrated that the TE-TRAP marker system provides a simple, rapid and cost-effective alternative for studying genetic diversity and identifying mutant lines in irradiated soybean mutant breeding.

## Figures and Tables

**Table 1:** Genetic diversity levels and polymorphic information content (PIC) values determined using three Class II DNA transposon markers in mutant diversity pool (MDP) soybean lines.

	MITE ^1^-Tourist	MITE-Stowaway	PONG
Numbers of fragments	139	162	106
Numbers of polymorphic fragments	73	98	66
Percentages of polymorphic fragments (%)	53.4	59.9	60.7
Numbers of monomorphic fragments	66	64	40
Percentages of monomorphic fragments (%)	47.6	40.1	39.3
PIC values	0.12	0.14	0.15

^1^ MITE: miniature inverted-repeat transposable element.

**Table 2:** Total and polymorphic fragment numbers, percentage of polymorphic fragments and polymorphism information content (PIC) values determined by each primer combination.

Primer Combination	Total Number of Fragments	Polymorphic Fragments	Polymorphism (%)	PIC
MITE ^1^-Stowaway + Sa4	39	15	38.46	0.09
MITE-Stowaway + Sa12	33	22	66.67	0.15
MITE-Stowaway + Ga3	32	21	65.63	0.17
MITE-Stowaway + Ga5	35	15	42.86	0.09
Total/Average	139	73	53.40	0.12
MITE-Tourist + Sa4	47	28	59.57	0.14
MITE-Tourist + Sa12	46	31	67.39	0.14
MITE-Tourist + Ga3	32	17	53.13	0.14
MITE-Tourist + Ga5	37	22	59.46	0.15
Total/Average	162	98	59.89	0.14
PONG + Sa4	31	24	74.42	0.20
PONG + Sa12	24	13	54.17	0.13
PONG + Ga3	29	16	55.17	0.15
PONG + Ga5	22	13	59.09	0.11
Total/Average	106	66	61.46	0.15
Total	407	237		
Average	33.92	19.75	58	0.14

^1^ MITE: miniature inverted-repeat transposable element.

**Table 3:** Analysis of molecular variance (AMOVA) of 208 soybean mutants using three Class II DNA transposon markers.

	Est. Var.	Percentage of Variation
PONG		
Among pop.	3.151	29%
Within pop.	7.646	71%
Total	10.797	100%
MITE ^1^-Stowaway		
Among pop.	2.209	20%
Within pop.	8.957	80%
Total	11.166	100%
MITE-Tourist		
Among pop.	2.766	18%
Within pop.	12.385	82%
Total	15.151	100%

^1^ MITE: miniature inverted-repeat transposable element.

**Table 4:** Sequences of fixed and arbitrary primers used to amplify TE-TRAP markers in MDP soybean lines.

Primer Name	Sequence (5′–3′)
Fixed primers	
MITE ^1^-Stowaway	CTT WTA DTT AGG GAY ARA GGG AG
MITE-Tourist	AAT TYT CTA TCC AAA CRC ACT C
PONG	AGA ARC CTG CAY TGG AGA TGC TC
Arbitrary primers	
Sa4	TTA CCT TGG TCA TAC AAC ATT
Sa12	TTC TAG GTA ATC CAA CAA CA
Ga3	TCA TCT CAA ACC ATC TAC AC
Ga5	GGA ACC AAA CAC ATG AAG A

^1^ MITE: miniature inverted-repeat transposable element.

## Data Availability

Not applicable.

## Supplementary Materials

The following are available online at https://www.mdpi.com/2223-7747/10/2/373/s1, Figure S1: Two-dimensional principal coordinates analysis ordination of MDP mutant lines based on TE-TRAP marker diversity. Figure S2: TEs sequence analysis and primer design progress flowchart.
