# Supplementary material for: Detecting Genetic Mobility Using a Transposon-Based Marker System in Gamma-Ray Irradiated Soybean Mutants
Source: Plants (Basel). 2021 Feb 15;10(2):373. doi: 10.3390/plants10020373 (PMC7919005; doi:10.3390/plants10020373)
Supplement: Supplementary file 1 [file plants-10-00373-s001.zip › plants-1087204-supplementary/Figure S1 Two-dimensional principal component analysis ordination of MDP mutant lines based on TE-TRAP marker diversity.docx]

(a)


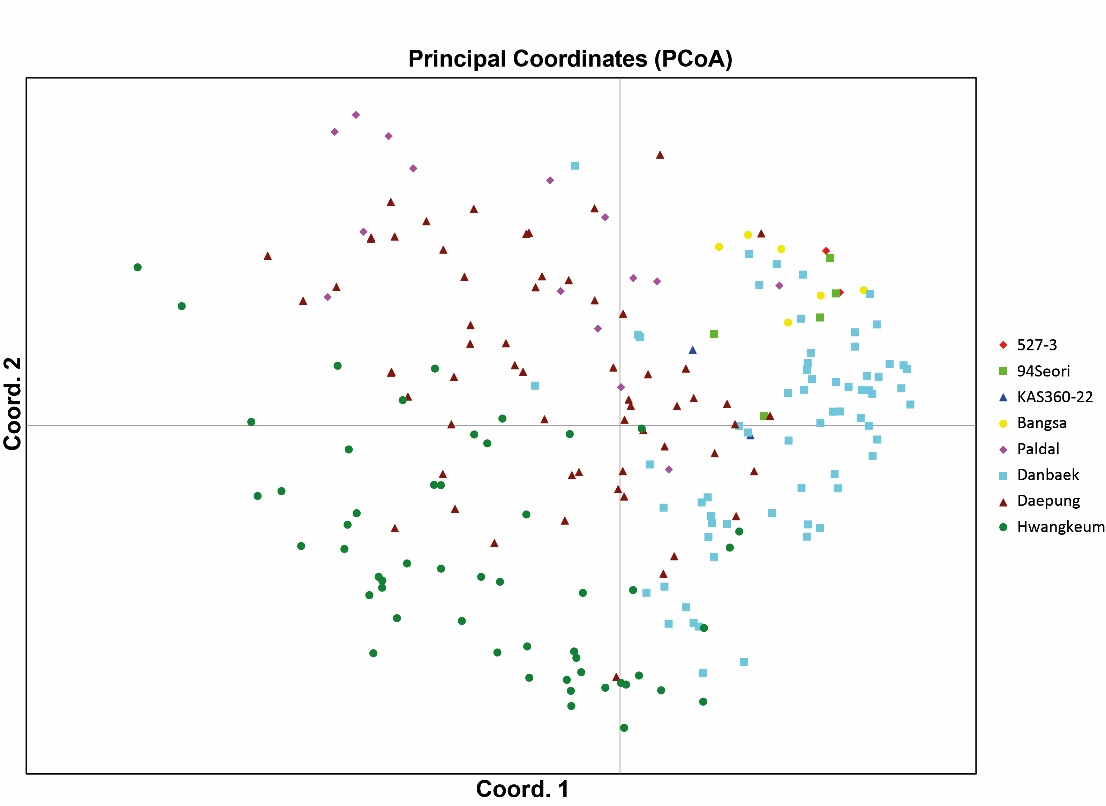


(b)


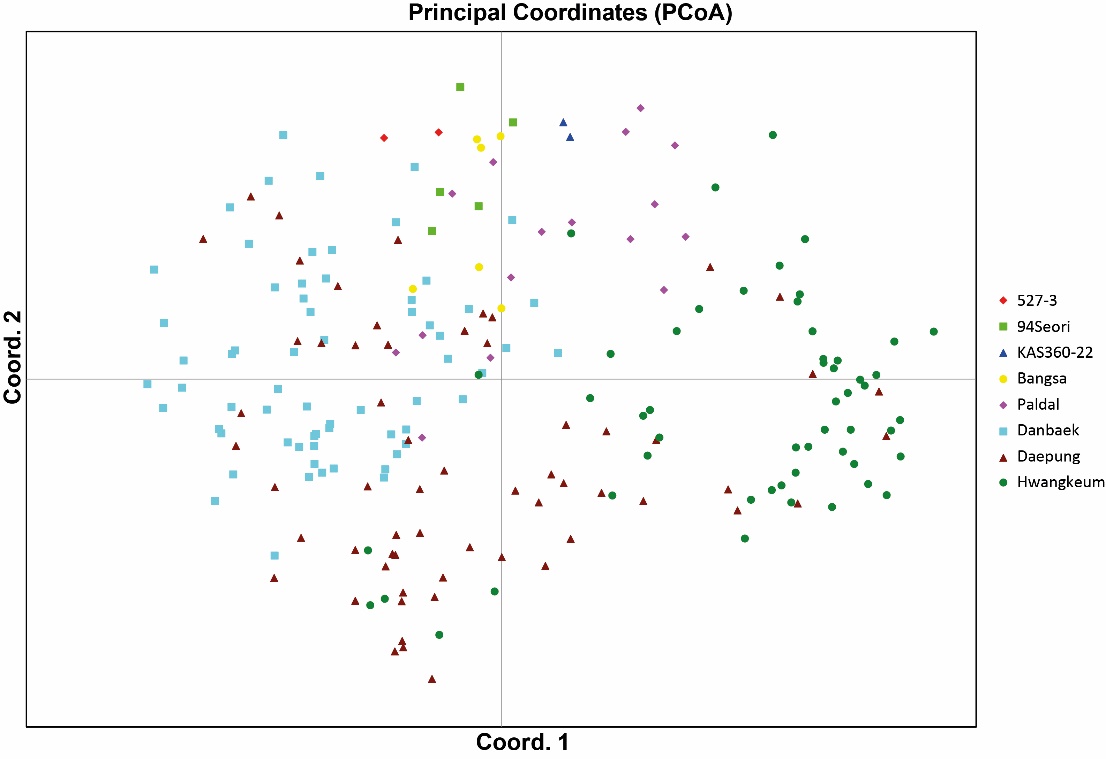


**Figure S1**: Two-dimensional principal component analysis ordination of MDP mutant lines based on TE-TRAP marker diversity (a) PCoA-MITE-Stowaway (b) PCoA-MITE-Tourist
