# Supplementary material for: Detecting Genetic Mobility Using a Transposon-Based Marker System in Gamma-Ray Irradiated Soybean Mutants
Source: Plants (Basel). 2021 Feb 15;10(2):373. doi: 10.3390/plants10020373 (PMC7919005; doi:10.3390/plants10020373)
Supplement: Supplementary file 1 [file plants-10-00373-s001.zip › plants-1087204-supplementary/Figure S2. Flowchart for primer designing.docx]

Obtained representative soybean MITE sequences from SoyTEdb: *Tourist, Stowaway, PIF/Pong*

Retrieved MITE groups with more than hundred members against BLAST program supporting in the SoyTEdb

(Aligned nucleotide sequences of TIR regions of each MITEs using the MAFFT program)

Design of degenerate primers from the conserved region covering all nucleotide sequences of each soybean MITEs

Design primer manually by software

**Figure S2**: TEs sequence analysis and primer design progress flowchart
